# Seed priming with selenium improves growth and yield of quinoa plants suffering drought

**DOI:** 10.1038/s41598-024-51371-6

**Published:** 2024-01-09

**Authors:** Muhammad Aown Sammar Raza, Muhammad Usman Aslam, Mohammad Valipour, Rashid Iqbal, Imran Haider, Abd El-Zaher M. A. Mustafa, Mohamed S. Elshikh, Iftikhar Ali, Rana Roy, Ayman M. S. Elshamly

**Affiliations:** 1 Department of Agronomy, The Islamia University of Bahawalpur, Bahawalpur, 63100 Pakistan; 2 Department of Engineering and Engineering Technology, Metropolitan State University of Denver, Denver, CO 80217 USA; 3 National Research Center of Intercropping, The Islamia University of Bahawalpur, Bahawalpur, 63100 Pakistan; 4 Department of Botany and Microbiology, College of Science, King Saud University, P.O. 2455, 11451 Riyadh, Saudi Arabia; 5 Department of Genetics and Development, Columbia University Irving Medical Center, New York, NY 10032 USA; 6 Institute of Plant Nutrition and Soil Science, Christian-Albrechts-Universität Zu Kiel, 24118 Kiel, Germany; 7 Department of Agroforestry & Environmental Science, Sylhet Agricultural University, Sylhet, 3100 Bangladesh; 8 Water Studies and Research Complex, National Water Research Center, Cairo, 81525 Egypt

**Keywords:** Biochemistry, Plant sciences

## Abstract

Drought stress is a worldwide threat to the productivity of crops, especially in arid and semi-arid zones of the world. In the present study, the effect of selenium (Se) seed priming on the yield of quinoa under normal and drought conditions was investigated. A pot trial was executed to enhance the drought tolerance in quinoa by Se seed priming (0, 3, 6, and 9 mg Se L^−1^). The plants were exposed to water stress at three different growth stages of quinoa, viz. multiple leaf, flowering, and seed filling. It was noticed that drought significantly affected the yield components of quinoa, however, Se priming improved the drought tolerance potential and yield of quinoa by maintaining the plant water status. Se priming significantly increased main panicle length (20.29%), main panicle weight (26.43%), and thousand grain weight (15.41%) as well as the gas exchange parameters (transpiration rate (29.74%), stomatal conductance (35.29%), and photosynthetic rate (28.79%), total phenolics (29.36%), leaf chlorophyll contents (35.97%), water relations (leaf relative water contents (14.55%), osmotic potential (10.32%), water potential (38.35%), and turgor potential (31.37%), and economic yield (35.99%) under drought stress. Moreover, Se priming markedly improved grain quality parameters i.e., phosphorus, potassium, and protein contents by 21.28%, 18.92%, and 15.04%, respectively. The principal component analysis connected the various study scales and showed the ability of physio-biochemical factors to describe yield fluctuations in response to Se seed priming under drought conditions. In conclusion, a drought at the seed-filling stage has a far more deleterious impact among other critical growth stages and seed priming with Se (6 mg L^−1^) was found more effective in alleviating the detrimental effects of drought on the grain yield of quinoa.

## Introduction

The world population is rapidly growing and proposed that it will reach to ~ 10 billion till 2050, demanding increase in food production to 50% over 2015 rates^1^. While the climate change is threating the food security mainly because of high temperature induced increasing drought episodes across the globe, as climate projection models proposed that 2.8 ± 0.4 °C temperature will be raised at the end of twenty-first century^2^. However, inclusion of climate resilient grain crops in the cropping pattern will help to solve this dilemma. In this vein, quinoa (*Chenopodium quinoa* Willd.) is an excellent choice which has the potential to withstand a wide range of abiotic stresses and grow effectively with minimal inputs. Its adaptation to drought, salinity and cold stresses, in addition to its marvelous nutritive value signifies quinoa as a climate-proof functional food^3,4^. Quinoa has exceptional nutritional properties like high protein content (15%), minerals (Mg, Zn, Fe, Cu, Ca), vitamins (B2, A, E) and all amino acids needed by a human body^5^. Antioxidant compounds like phytosterols, polyphenols and flavonoids with possible nutraceutical benefits are among dietary merits of quinoa^6^. Quinoa contains up to 8.8% fat, 4.2% ash, 60.1% starch and 3.4% crude fiber^7^. The successful cultivation of quinoa in Pakistan has led to comprehensive exploration of its growth and production potential^8^. However, it is important to note that quinoa growth and yield can be significantly affected under severe drought stress (DS)^9–11^. Particulalrly, the flowering and seed filling stages have been found as the most sensitive and critical stages of quinoa to DS, ultimately limiting crop yield^11^.

Drought significantly hampered the plant growth and development, resulting in a notable decrease in biomass accumulation and crop growth rate^12^. The primary consequences of drought on crop plants include a reduction in the leaf size, rate of cell division, root proliferation and stem elongation^10,11^. Drought also distrupts stomatal oscillations and affects plant water and nutrient relations, water use efficiency (WUE) and crop productivity^13^. Under DS conditions, an unnecessary rise in reactive oxygen species (ROS) causes oxidative damage and, ultimately, plant death^14^. Seed priming is the controlled hydration and drying of seeds in order to indorse rapid germination and sustained establishment under stress conditions^15^.

Selenium (Se) has been described as a vital nutrient for humans, animals and plants, as well as an environmental toxin^16^. The threshold of Se toxicity and deficiency is insignificant and depends on factors such as its chemical composition, concentration and various environmental conditions^17^. Se is a beneficial micronutrient that functions as an anti-senescent, antioxidant, and involved in plant’s active defense against biotic and abiotic stresses^18,19^. The application of Se has the ability to improve the crop's nutritional value, economic yield, and plant abiotic tolerance. Se enhances crop growth by the accumulation of starch in the chloroplast^20^. Moreover, Se can control the activity of a number of antioxidant enzymes and metabolites, enabling plants to withstand oxidative stress^21^. However, Se toxicity or benefits depend greatly on the concentration used^17^.

Drought, a multi-dimensional stress affects various physiological and biochemical attributes in plants including photosynthetic rate, turgor potential, osmotic potential along with severe injury to cellular membranes^22,23^. Plants accumulate active oxygen species such as OH, O_2_^−^ and H_2_O_2_ in response to DS^24^. The cell systems of plants are protected from the cytotoxic effect of these active radicals by enzymatic activity and non-enzymatic antioxidants^25–27^. However, the protective role of foliar Se application has also been reported under DS conditions. Tadina et al.^28^ observed a significantly lower stomatal conductance in water deficient plants of common buckwheat as compared to Se treated water deficient plants with higher stomatal conductance. Se application as Na_2_SeO_4_ considerably increased root activity, proline contents, chlorophyll contents, photosynthetic rate and grain yield under abiotic stress conditions in *Sorghum bicolor* L.^18^. Se priming and foliar application significantly improved the osmotic potential, water potential, turgor potential, total chlorophyll contents, biological yield, and grain yield in camelina and canola crops under DS^29^. Furthermore, Se application vicissitudes the membrane activity and permeability and acts as a cofactor for the glutathione peroxidase enzyme that catalyses the peroxide reduction reaction, protects plants from DS and offsets oxidative stress by impeding lipid peroxidation^30^.

The study focused on investigating the effectiveness of Se seed priming in enhancig the drought tolerance potential of quinoa by improving its physiological and biochemical attributes. One of the key objective of the study was to assess the impact of Se seed priming on the growth, yield, and physiological attributes of quinoa subjected to drought stress conditions.

## Materials and methods

### Layout and crop management

**Figure 1:**
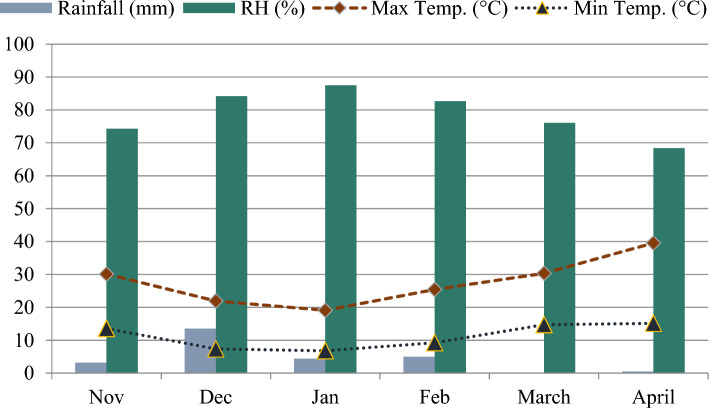
Relative humidity (RH), rainfall, maximum and minimum temperature during the growing period of quinoa.

The experiment was completed in the wirehouse of the Department of Agronomy, The Islamia University of Bahawalpur, Pakistan (Longitude: 71° 40′ 59.99″ E; Latitude: 29° 23′ 60.00″ N). The physio-chemical examination of experimental soil was performed before sowing and is given in Table 1. Relative humidity (RH), rainfall, maximum and minimum temperature (digital thermometer (Youshiko YC9070) data throughout the growing season of quinoa is depicted in Fig. 1. The quinoa seeds (UAF-Q7) were collected from the University of Agriculture, Faisalabad. The experiment was set up using a completely randomized design (CRD) in factorial arrangement, with four repetitions in plastic pots measuring 30 × 30 × 23 cm and containing 17.5 kg of soil each. These pots were set in a wirehouse, over which a clear plastic sheet could be draped to protect crop plants from rainfall, whenever necessary. Ten quinoa seeds per pot were planted and four plants per pot were maintained by removing extra plants at two leaf stage. To minimize any ambiance-related impacts, pot positions were rotated every week. Each pot received a fertilizer application of 2 g N and 1.5 g P_2_O_5_ per pot. The entire amount of phosphorus and nitrogen was used as a basal dose.

**Table 1:** Physiochemical properties of the experimental soil.

Parameters	Soil profile
Clay (%)	9.5
Silt (%)	33.5
Sand (%)	57
pH	7.37
Texture class	Sandy loam soil
Organic matter (%)	0.91
Electric conductivity (dS m^−1^)	2.51
Available Potassium (ppm)	109
Ammoniac N (mg g^−1^)	1.62
Available Phosphorus (ppm)	6.77

### Drought imposition and Se priming

Drought stress was executed at multiple leaf (MLS) 30 days after germination (DAG), flowering (FS, 60 DAG) and seed filling (SFS, 85 DAG) stages, whereas, normal supply of water was used as control treatment (CK). Quinoa plants were examined everyday to reveal their phenology for precise application of growth stage-based drought stress (DS) outlined by Sosa-Zuniga et al.^31^. All pots received the same amount of water at 80% of soil water holding capacity (WHC), also reffered as control treatment, until the commencement of DS. After that, DS was enacted at MLS, FS and SFS by maintaining the soil WHC at 25%.

Se as Sodium selenate (Na_2_SeO_4_) was used for seed priming. The solution of 3.0, 6.0 and 9.0 mg L^−1^ Se was prepared by soaking quinoa seeds in Na_2_SeO_4_ solution for 6 h and then dried under shade until their original moisture level before sowing.

### Data recording and related procedures

#### Growth and yield related parameters

The plants were harvested 140 DAS when they reached at maturity in order to measure plant height, number of panicles per plant, length and weight of main panicle, thousand grain weight, biological and economic yield.

#### Physiological attributes

The chlorophyll contents of leaves were assessed from fully expanded young leaves after 50 DAS using a chlorophyll meter (model CL-01, Hansatech instruments Ltd., United Kingdom), and the calculation of water use efficiency (WUE) was based on the specified formula described by Iqbal et al.^32^, WUE = grain yield/total water applied. The determination of total phenolics in leaves followed the standard protocol outlined by Waterhouse^33^.

The gas exchange attributes i.e., transpiration rate, photosynthetic rate and stomatal conductance, of fifth topmost fully expanded young leaves were measured using LI-COR Li-6400 (USA) portable photosynthetic system, at noon.

#### Water relations

The leaf water potential and relative water contents (RWC) were assessed using two completely expanded fresh leaves after fifty days of germination,. PSYPRO thermocouple psychrometer (Wescor, USA) was used to measure the leaf water potential. Fresh leaves were removed from plants and instantly shifted to test site (laboratory) to record fresh weight (FW). Then, the leaves were dipped in distilled water for 24 h at 4 °C and their turgid weight (TW) was measured. The dry weight of the leaves was then noted by placing them in an oven set at 70 °C till constant weight. Leaf RWC (%) were determined by using the given formula^34^.

$${\text{RWC}}\left( \% \right) = \left( {{\text{FW}} - {\text{DW}}} \right)/\left( {{\text{TW}} - {\text{DW}}} \right) \times {1}00$$

Water potential apparatus (Chas W. Cook, Birmingham, England) was used to record the leaf water potential (− MPa, LWP). Leaf osmotic potential (− MPa, LOP), was computed by freezing the leaves in liquid nitrogen, after that the sap was extracted out to quantify the osmotic potential through a vapor pressure osmometer (Wescor 5520, Logan, USA). Leaf turgor potential (MPa, LTP) was calculated by following formula^35^.

$${\text{LTP}} = {\text{LWP}} - {\text{LOP}}$$

#### Quality parameters

Quinoa grains (0.1 g) were ground into dry powder and placed in digestion tubes. To each tube, 5 ml of rigorous H_2_SO_4_ was added, and the digestion tubes were incubated at 25 °C overnight. Subsequently, one ml of 34% H_2_O_2_ was carefully added to the digestion tube. These tubes were then positioned in a digestion block and heated at 350 °C until fumes formation and heating was continued to further for thirty minutes. The digestion tubes were taken out of the block and allowed to cool. The tubes were then reinserted into the digestion block after the addition of 1 ml of H_2_O_2_. The exceeding step was repeated till the cooled digested substance become colorless. The extract was diluted to 50 ml in flasks, sieved and used to determine the nitrogen concentration using Kjeldahl’s method and nitrogen (N) content was then multiplied with a factor 6.25 to get the protein content (%)^36,37^. The phosphorus and potassium content in the digests were ascetained by using spectrophotometer (Shimadzu, UV-1201, Kyoto, Japan) at λ = 820 nm and flame photometer (PFP-7, Jenway, UK), as described by Rosero et al.^38^ and Chapman and Pratt^39^, respectively.

### Statistical analysis

The data gathered for all attributes were analyzed statistically by Fisher’s analysis of variance techniques. LSD (least significant difference) test was carried out at 5% probability level to evaluate the differences between the treatment means using the STATISTIX software (version 8.1)^40^. The coorelation among grain yield, grain weight and main panicle length and physiobiochemical attributes were investigated using multivariate analysis. Principal component analysis (PCA) was performed on the data and the results were visualised with biplot graph settled from PC1 and PC2. The software Origin Pro 9.1 (Origin-Lab Corporation, Northampton, MA) was used to create the figures.

### Ethics approval and consent to participate

This study does not include human or animal subjects.

### Statement on guidelines

All experimental studies and experimental materials involved in this research are in full compliance with relevant institutional, national and international guidelines and legislation.

## Results

### Growth and yield attributes

Drought stress significantly affected the biomass production and yield attributes [number of panicles per plant (NPPP), main panicle length (MPL), main panicle weight (MPW) and thousand grain weight (TGW) in quinoa (Table 2). Priming of quinoa seeds with Se mitigated the negative effects of drought showing the maximum values for Se_2_ (6.0 mg L^−1^) and minimum values in control treatment (no priming) for all growth and yield contributing traits. However, quinoa genotype responded differently to DS imposition at critical growth stages (CGS). Highest values for all studied parameters were observed under normal irrigation (Ck) while lowest values for NPPP, MPL, MPW and TGW were recorded in Ck, DMLS, DFS and DSFS, respectively. Furthermore, seed priming with 6.0 mg L^−1^ Se was found best among other treatments showing maximum GY (16.67) under Ck and minimum value (10.67) was observed in DSFS.

**Table 2:** Plant height (PH, cm), panicles per plant (PPP), main panicle length (MPL, cm), main panicle weight (MPW, g), thousand grain weight (TGW, g), biological yield (BY, g plant^−1^) and grain yield (GY, g plant^−1^) affected by Se priming under DS conditions.

Treatments	PH	PPP	MPL	MPW	TGW	BY	GY
Ck							
Se_0_ (Control)	109.32 ± 5.36 cd	11.12 ± 2.11 bc	17.00 ± 2.53 cde	21.47 ± 3.01 abc	2.75 ± 0.27 bcd	39.11 ± 7.85 bc	15.67 ± 3.28 abc
Se_1_ (3.0 mg L^−1^)	113.27 ± 5.32 a	11.89 ± 2.04 ab	18.24 ± 2.34 a	22.48 ± 3.06 a	2.85 ± 0.26 ab	41.92 ± 7.16 ab	16.00 ± 2.96 ab
Se_2_ (6.0 mg L^−1^)	114.44 ± 3.91 a	12.80 ± 2.01 a	18.53 ± 2.11 a	23.08 ± 3.52 a	2.92 ± 0.26 a	43.85 ± 7.82 a	16.67 ± 3.46 a
Se_3_ (9.0 mg L^−1^)	112.33 ± 5.52 ab	11.50 ± 2.09 b	17.99 ± 2.11 ab	22.14 ± 3.15 ab	2.79 ± 0.25 abc	40.52 ± 7.46 ab	15.33 ± 3.48 bcd
DMLS							
Se_0_ (Control)	106.30 ± 6.12 f.	8.73 ± 2.24 h	15.90 ± 2.71 ghi	19.64 ± 4.21 de	2.62 ± 0.35 defg	30.28 ± 9.74 g	14.33 ± 3.46 de
Se_1_ (3.0 mg L^−1^)	106.76 ± 6.61 ef	9.13 ± 2.59 fgh	17.46 ± 2.68 bc	20.69 ± 3.99 bcd	2.75 ± 0.35 bcd	31.39 ± 8.74 fg	14.67 ± 4.06 cd
Se_2_ (6.0 mg L^−1^)	107.08 ± 6.36 def	9.35 ± 2.71 efgh	17.86 ± 2.36 ab	22.47 ± 3.75 a	2.76 ± 0.30 bcd	32.58 ± 9.49 fg	15.67 ± 3.76 abc
Se_3_ (9.0 mg L^−1^)	106.11 ± 6.24 f.	8.65 ± 2.48 h	17.15 ± 2.48 cd	20.30 ± 3.92 cde	2.70 ± 0.30 cde	30.47 ± 9.05 g	14.33 ± 3.76 de
DFS							
Se_0_ (Control)	108.24 ± 5.87 cdef	9.05 ± 2.69 gh	14.77 ± 2.62 k	17.31 ± 4.45 gh	2.48 ± 0.37 gh	34.72 ± 9.99 def	11.00 ± 4.06 hi
Se_1_ (3.0 mg L^−1^)	108.74 ± 6.43 cde	9.78 ± 2.61 efg	15.31 ± 2.74 ijk	19.35 ± 3.07 def	2.60 ± 0.35 efgh	38.95 ± 8.26 bc	13.33 ± 3.48 ef
Se_2_ (6.0 mg L^−1^)	109.19 ± 6.25 cd	10.17 ± 2.82 cde	15.60 ± 2.38 hij	19.78 ± 3.74 de	2.65 ± 0.33 cdef	40.82 ± 9.43 ab	14.33 ± 3.75 de
Se_3_ (9.0 mg L^−1^)	108.13 ± 5.17 cdef	9.50 ± 2.53 efgh	14.95 ± 2.59 jk	18.77 ± 4.13 efg	2.58 ± 0.31 efgh	37.08 ± 9.30 cd	13.00 ± 4.04 fg
DSFS							
Se_0_ (Control)	109.18 ± 5.76 cd	10.06 ± 2.05 def	14.88 ± 2.76 k	16.98 ± 4.07 h	2.47 ± 0.35 h	30.65 ± 10.27 g	10.67 ± 3.75 i
Se_1_ (3.0 mg L^−1^)	110.39 ± 6.66 bc	11.22 ± 2.29 b	16.39 ± 2.61 efg	17.33 ± 3.85 gh	2.49 ± 0.33 gh	34.74 ± 9.14 def	11.67 ± 3.75 hi
Se_2_ (6.0 mg L^−1^)	113.01 ± 6.22 a	11.75 ± 2.52 b	16.74 ± 2.25 def	17.97 ± 3.74 fgh	2.52 ± 0.31 fgh	36.71 ± 9.61 cde	12.00 ± 4.04 gh
Se_3_ (9.0 mg L^−1^)	110.27 ± 6.39 bc	10.95 ± 2.45 bcd	16.06 ± 2.47 fgh	17.14 ± 3.69 gh	2.49 ± 0.27 gh	33.40 ± 8.75 efg	11.00 ± 4.04 hi
LSD (p ≤ 0.05)	*2.28*	*1.01*	*0.69*	*1.63*	*0.14*	*3.43*	*1.24*
Significance							
Se	**	**	**	**	**	**	**
Drought	**	**	**	**	**	**	**
Se × Drought	ns	ns	ns	ns	ns	ns	ns

Asterisks (*) indicate significant differences; ∗P ≤ 0.05, ∗∗P ≤ 0.01; ns, non-significant. Ck, DMLS, DFS, and DSFS represents control, drought at multiple leaf, flowering, and seed filling stages, correspondingly. Mean values are presented with standard error, SE (n = 4). Means that share the same letter case are not substantially different at 5% probability level.

Significant values are in italics.

### Leaf chlorophyll contents

Leaf chlorophyll contents (LCC) of quinoa were affected significantly by the execution of DS as presented in Table 3. The peak values for LCC (22.52) were observed when crop was nourished with normal irrigation (Ck) followed by DSFS and minimum LCC (14.55) were observed when crop was subjected to drought at MLS. Se priming significantly improved LCC showing maximum value for 6.0 mg L^−1^ Se and lowest LCC were noted in control treatment (Se_0_).

**Table 3:** Protein contents (PC, mg g^−1^), phosphorus contents (P, mg g^−1^) potassium contents (K, mg g^−1^), leaf chlorophyll content (mg g^−1^ fresh weight, LCC), water use efficiency (kg ha^−1^ mm^−1^, WUE) and total phenolics (mg g^−1^ fresh weight, TP) affected by Se priming under DS conditions.

Treatments	PC	P	K	LCC	WUE	TP
Ck						
Se_0_ (Control)	13.27 ± 1.06 h	347.93 ± 73. 05 cdef	1064.0 ± 126.79 abcd	20.08 ± 4.33 bcd	0.39 ± 0.07 ef	10.07 ± 1.99 cd
Se_1_ (3.0 mg L^−1^)	13.34 ± 0.99 h	377.10 ± 53.78 ab	1070.1 ± 139.82 abcd	21.74 ± 4.05 ab	0.40 ± 0.11 def	10.67 ± 1.87 abc
Se_2_ (6.0 mg L^−1^)	13.87 ± 1.05 gh	380.48 ± 58.52 ab	1114.1 ± 107.33 ab	22.52 ± 4.41 a	0.42 ± 0.12 def	11.02 ± 1.48 ab
Se_3_ (9.0 mg L^−1^)	13.30 ± 1.15 h	395.16 ± 63.25 a	1129.5 ± 123.53 a	20.45 ± 3.93 abc	0.38 ± 0.12 f.	11.41 ± 1.81 a
DMLS						
Se_0_ (Control)	13.32 ± 1.22 h	346.70 ± 65.65 cdef	986.8 ± 175.38 defg	14.42 ± 5.52 i	0.51 ± 0.07 abc	8.06 ± 2.37 i
Se_1_ (3.0 mg L^−1^)	14.10 ± 1.29 efg	356.79 ± 70.52 bcde	1032.2 ± 177.95 bcde	14.91 ± 4.66 hi	0.52 ± 0.14 ab	8.10 ± 2.03 hi
Se_2_ (6.0 mg L^−1^)	14.36 ± 1.38 defg	363.75 ± 76.74 bc	1049.8 ± 126.58 abcde	16.85 ± 5.21 fgh	0.56 ± 0.13 a	8.31 ± 2.16 ghi
Se_3_ (9.0 mg L^−1^)	13.99 ± 1.64 fg	370.49 ± 71.67 abc	1090.8 ± 126.08 abc	14.55 ± 5.33 i	0.51 ± 0.12 abc	8.49 ± 2.33 fghi
DFS						
Se_0_ (Control)	14.27 ± 1.09 defg	311.95 ± 58.23 g	933.2 ± 158.27 g	14.83 ± 5.42 hi	0.39 ± 0.09 ef	8.34 ± 2.49 ghi
Se_1_ (3.0 mg L^−1^)	14.74 ± 1.26 cde	323.98 ± 69.53 fg	1020.5 ± 163.85 cdef	17.27 ± 4.44 fg	0.47 ± 0.12 bcd	8.64 ± 2.14 fghi
Se_2_ (6.0 mg L^−1^)	15.02 ± 1.32 abc	332.47 ± 75.09 efg	1046.3 ± 117.46 abcde	18.02 ± 5.02 defg	0.51 ± 0.13 abc	9.12 ± 2.42 efg
Se_3_ (9.0 mg L^−1^)	14.53 ± 1.22 cdef	361.39 ± 73.28 bcd	1081.8 ± 141.89 abc	16.32 ± 5.33 ghi	0.46 ± 0.14 bcde	9.32 ± 2.49 def
DSFS						
Se_0_ (Control)	14.76 ± 1.44 bcd	311.07 ± 63.02 g	915.8 ± 145.61 g	17.59 ± 5.18 efg	0.39 ± 0.08 ef	9.00 ± 2.33 efgh
Se_1_ (3.0 mg L^−1^)	15.40 ± 1.37 ab	314.24 ± 66.86 g	945.1 ± 155.23 fg	19.52 ± 3.27 cde	0.43 ± 0.13 def	9.87 ± 2.04 cde
Se_2_ (6.0 mg L^−1^)	15.62 ± 1.33 a	321.08 ± 56.09 fg	938.2 ± 108.19 fg	20.27 ± 4.71 bc	0.44 ± 0.14 cdef	10.10 ± 2.31 bcd
Se_3_ (9.0 mg L^−1^)	15.07 ± 1.64 abc	334.52 ± 76.03 defg	967.6 ± 138.04 efg	18.81 ± 4.88 cdef	0.40 ± 0.13 def	10.41 ± 2.35 bc
LSD (p ≤ 0.05)	*0.64*	*28.11*	*85.81*	*2.06*	*0.08*	*0.93*
Significance						
Se	**	**	**	**	**	*
Drought	**	**	**	**	**	**
Se × Drought	ns	ns	ns	ns	ns	ns

Asterisks (*) indicate significant differences; ∗P ≤ 0.05, ∗∗P ≤ 0.01; ns, non-significant. Ck, DMLS, DFS, and DSFS represents control, drought at multiple leaf, flowering, and seed filling stages, correspondingly. Mean values are presented with standard error, SE (n = 4). Means that share the same letter case are not substantially different at 5% probability level.

Significant values are in italics.

### Water use efficiency

Drought and Se priming significantly influenced the WUE of quinoa. Maximum values (0.56) for WUE were noted in DMLS and minimum value (0.38) for WUE was found in Ck. Highest WUE was noticed when quinoa seeds were primed with 6.0 mg L^−1^ Se (Se_2_) and minimum was found in Se_0_ (Table 3).

### Total phenolics

Total phenolics (TP) were significantly affected by both deficit irrigation and Se priming in quinoa (Table 3). Maximum TP were obtained in control treatment (Ck) whereas minimum reading was noted for TP in DMLS treatment. Se significantly alleviated the adverse effects of drought and improved the accumulation of TP in quinoa leaves. Maximum TP (11.41) were recorded in Se_3_ (9.0 mg L^−1^) and minimum TP (8.06) were recorded in control treatment (Se_0_).

### Gas exchange parameters

**Figure 2:**
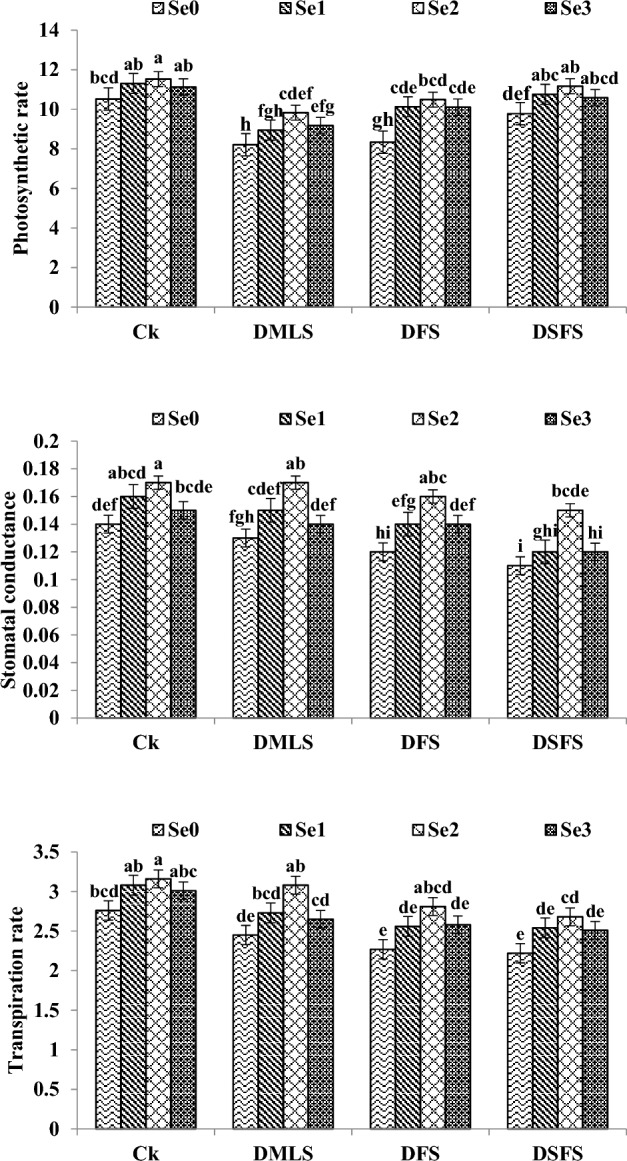
Photosynthetic rate (µmol m^−2^ s^−1^), Stomatal conductance (mol m^−2^ s^−1^) and transpiration rate (mmol m^−2^ s^−1^) of quinoa affected by Se priming under drought. Se_0_, Se_1_, Se_2_ and Se_3_ indicates 0, 3.0, 6.0 and 9.0 mg L^−1^ Se. Ck, DMLS, DFS, and DSFS represents control, drought at multiple leaf, flowering, and seed filling stages, correspondingly. The error bars represent standard error (n = 4).

The quinoa plants subjected to DS decreased the photosynthetic rate (PR), stomatal conductance (SC) and transpiration rate (TR) as compared to control plants (Fig. 2). Highest values for PR were observed in control treatment followed by DSFS and DFS and minimum PR was achieved in DMLS. A disparate fashion (Ck > DMLS > DFS > DSFS) was observed for SC and TR under drought. Se seed priming significantly improved the PR, SC and TR in DS plants as compared to control treatment. Maximum values for PR (11.53), SC (0.17) and TR (3.16) were achieved when quinoa seeds were primed with 6.0 mg L^−1^ Se (Se_2_) followed by Se_1_ and Se_3_ both under control and drought stressed plants.

### Water relations

**Figure 3:**
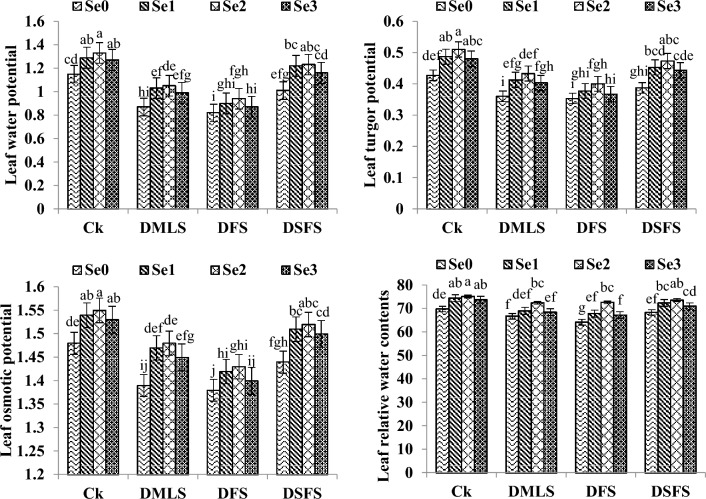
Leaf water potential (− MPa), Leaf turgor potential (MPa), Leaf osmotic potential (− MPa) and Leaf relative water contents (%) of quinoa influenced by Se priming under drought. Se_0_, Se_1_, Se_2_ and Se_3_ indicates 0, 3.0, 6.0 and 9.0 mg L^−1^ Se. Ck, DMLS, DFS, and DSFS represents control, drought at multiple leaf, flowering, and seed filling stages, correspondingly. The error bars represent standard error (n = 4).

Significant effect of drought and Se seed priming on LWP, LTP, LOP and LRWC has been presented in Fig. 3. Significant reduction in LWP, LTP, LOP and LRWC was observed in DSFS treatment followed by DMLS and DFS when compared with non-stressed quinoa plants. Se seed priming significantly increased the LWP, LTP and LOP in comparison with control treatment. Furthermore, Se priming (6.0 mg L^−1^) substantially improved the aforementioned parameters as compared to control treatment both under well watered and drought conditions.

### Grain protein contents

Table 3 indicates that drought has significant effect on protein contents (PCs) of quinoa grains. Maximum values for PCs were noted by the execution of drought at SFS followed by FS and MLS and minimum value was noted in Ck. Maximum grain PCs (15.62) were observed in Se_2_ treatment (6.0 mg L^−1^) while reduction in PCs (13.27) was recorded in control (Se_0_) when no seed priming was done. The interaction of both Se and drought non-significantly affected the grain PCs of quinoa.

### Grain phosphorus contents

Irrigation deficit and seed priming with Se influenced significantly the grain phosphorus contents of quinoa. Among the drought maximum grain phosphorus contents were under normal irrigation (D_0_) followed by D_1_ and minimum grain phosphorus contents were found in D_3_ which was at par with D_2_. For Se, maximum phosphorus contents (395.16) in grains were recorded in Se_3_ and reduction (311.07) in the said parameter was noticed in control treatment (Se_0_) which was statistically at par with Se_1_ (Table 3).

### Grain potassium contents

Irrigation deficit and seed priming with Se influenced significantly the potassium (K) contents in quinoa grains. Maximum K (1129.5) values in quinoa seeds were found with normal irrigation (Ck) followed by DMLS and lower values for K content (915.8) were noted in DSFS treatment. Seed priming with Se significantly improved the concentration of K in quinoa grains both under Ck and water deficit conditions (Table 3).

### Principle component analysis (PCA) for data mining

**Figure 4:**
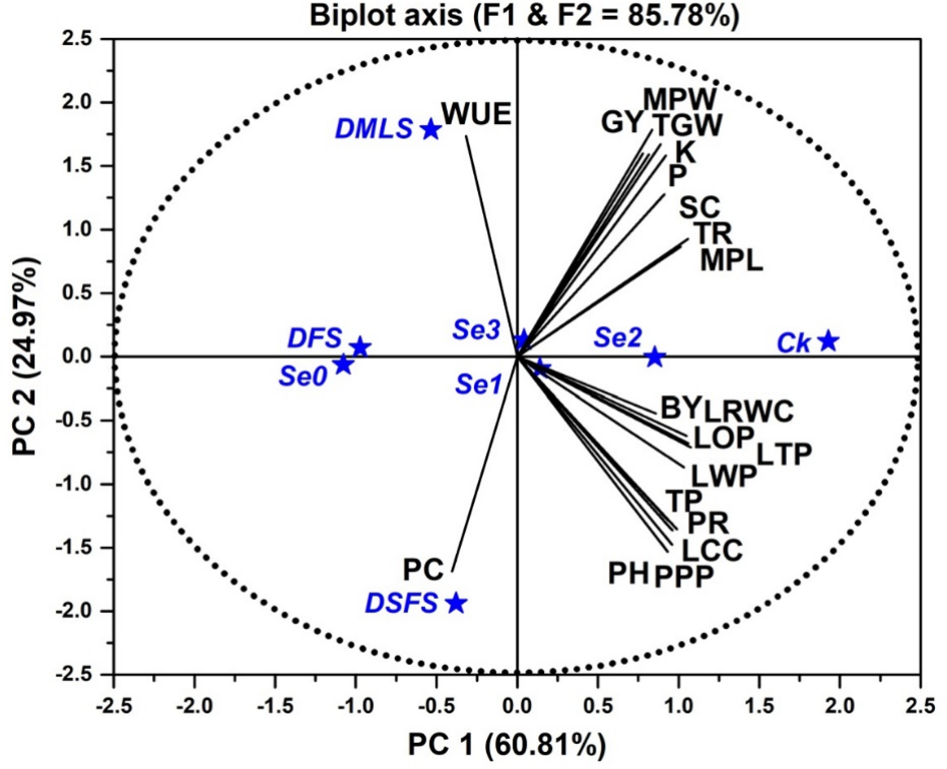
Biplot showing the correlations between plant height (PH), panicles per plant (PPP), main panicle length (MPL), main panicle weight (MPW), thousand grain weight (TGW), biological yield (BY), grain yield (GY), protein contents (PC), phosphorus contents (P) potassium contents (K), leaf chlorophyll content (LCC), total phenolics (TP), water use efficiency (WUE), photosynthetic rate (PR), stomatal conductance (SC), transpiration rate (TR), leaf water potential (LWP), leaf turgor potential (LTP), leaf osmotic potential (LOP) and leaf relative water contents (LRWC) of quinoa affected by Se priming under drought. Se_0_, Se_1_, Se_2_ and Se_3_ indicates 0, 3.0, 6.0 and 9.0 mg L^−1^ Se and Ck, DMLS, DFS, and DSFS represents control, drought at multiple leaf, flowering, and seed filling stages, correspondingly.

The PCA was executed on all experimental parameters to reveal their correlation with the neighboring treatments and other variables. Yet, the PCA revealed a clear distinction between PC1 and PC2, which accounted for a combined 85.78% of the total variability in grain yield of quinoa and associated parameters with water relations. Figure 4 indicates that PCA loading and score plot concede that PC1 and PC2 were responsible for 60.81% and 24.97% of the total variance, respectively. The cosine angle amongst two trait vectors signposts association between traits, whereas, acute and obtuse angles shows a negative and positive correlation, respectively, whereas the right angle among two trajectories of variables demonstrates no association among them. Biplot graph from PCA unfolds several significant associations such as a positive association between the grain yield (GY) and its relted traits (PPP, MPL, MPW, TGW) as well as a strong correlation between GY and water-related (WUE, LRWC, LWP, LOP, LTP) and gas exchange (PR, SC, TR) attributes.

## Discussion

Drought is one of the main limiting factor to achieve sustainability in crop production^41,42^. A considerable reduction in the growth and yield of quinoa under drought conditions demands for the development and identification of drought alleviation strategies. The outcomes of present study showed that priming of quinoa seeds with Seinfluenced significantly the growth, yield and its components both under control and moisture deficit conditions. Our findings support preceding studies who reported that Se priming could be an effective tool in ameliorating the DS for increasing wheat growth and yield under controlled conditions^43,44^.

A significant decrease in photosynthetic pigment (chlorophyll) was due to the production of reactive oxygen species (ROS) such as H_2_O_2_ and O_2_ leading to lipid peroxidation and consequently, chlorophyll destruction^45^, which ultimately resulted in demolition of chlorophyll under stress situations. However, seed priming with Se resulted in higher chlorophyll contents both in drought and non-stressed environments because Se increased the flow of electrons in the respiratory chain, and hence increased the rate of photosynthesis in plants^46^. Similar conclusions were drawn by others that Se priming improved the leaf chlorophyll contents in goji berry^47^ kenaf^48^ and lettuce^49^. Higher concentrations of Se decreased chlorophyll content in basil plants^50^ and rice seedlings^51^ which support our findings.

Drought, when imposed at FS and SFS negatively affected WUE of quinoa. Analogous results have been documented by Zhang et al.^52^ and Geerts et al.^53^ for wheat and quinoa, correspondingly, under drought conditions. Similarly, Telahigue et al.^54^ and Aslam et al.^8^ reported results in-line with our findings that WUE generally increased under poor water supply in quinoa. Se seed priming improved the WUE both in DS and control treatment, possibly due to the better accessibility of water to quinoa plants. Similarly, Sajedi et al.^55^ also described a significant improvement in WUE of maize plants by Se application. Figure 4 indicates that WUE is a significant determinant for quinoa yield vacillations at CGS.

Drought stress significantly abridged the water relations (LWP, LTP, LOP and LRWC) in quinoa when compared with control treatment. Similar outcomes have been stated by Haider et al.^56^ in wheat and Iqbal et al.^32^ in cotton that DS decreased all these parameters. This decrease could be brought about by inadequate food absorption, decreased cell division, SC, and less cytokines production^57^. An increase in the aforementioned characteristics was seen when quinoa seeds were primed with Se. Likewise, Ahmad et al.^29^ observed that Se priming improves the water uptake without decreasing the transpiration rate in oilseed crops that helps to increase LRWC, OP and TP. Domokos-Szabolcsy et al.^58^ revealed that Se seed treatment reinforce the defense mechanism of plants against DS by improving transfer of electrons and the activity of protective enzymes, thus curtailing the damage caused by DS to photosynthetic apparatus.

Drought stress significantly decreased phenolic content of quinoa when imposed at MLS which may be due to the reduced activity of plant hormones (phenylalaine ammonia lyase) and osmotic stress during the biosynthesis of phenolic compounds. Similarly, Moharramnejad et al.^59^ and Sofy et al.^60^ reported the reduction of phenolics accumulation in maize and *Hordeum vulgare* under DS. In contrary, Salem et al.^61^ and Kusvuran and Dasgan^62^ described an increase in phenolics under irrigation deficit. Se priming significantly increased leaf phenolic contents both in drought and irrigated control. Similarly, an increase in total phenolic contents has been reported in rice by the application of Se^51,63^.

Protein contents (PCs) in quinoa grains were considerably improved when DS was executed at SFS. Anjum et al.^64^ also reported similar findings that PCs increased under drought conditions in maize. Se supplementation enhanced the accretion of PCs in quinoa both under control and water deficit conditions but plants emerged from the seeds primed with higher concentration of Se showed a decrease in PCs. Similarly, Nawaz et al.^65^ concluded that Se priming enhanced crude protein in drought stressed maize plants. Our findings are in accordance with the above stated results that PCs were increased with Se priming.

Drought stress significantly reduced the concentration of P and K in quinoa seeds when drought was imposed at DSFS which may be because of the inability of plant roots to uptake soil nutrients present in soil and their upward movement within plants. Similarly, Jin et al.^66^ found that uptake and translocation of P from roots to seeds of soybean was reduced under DS and Nawaz et al.^67^ reported a decrease in potassium uptake under late DS in wheat. The priming of quinoa seeds with Se demonstrated beneficial effects in counteracting the effects of drought by enhancing the level of P and K in seeds both under drought and control conditions. Hajiboland et al.^68^ stated similar results that higher level of Se application enhanced P and K concentration in wheat.

The yield contributing attributes (NPPP, MPL, MPW, TGW), biological yield (BY) and grain yield (GY) were lowered under irrigation deficit. However, decrease in NPPP and BY at MLS can be attributed to limited N and P absorption in the begining of plant growth^67^. The aforementioned yield components were reduced when moisture stress was given at DSFS except MPL showing minimum values at DFS which might be due to the inadequate absorption and distribution of photosynthates. Water stress in quinoa at DSFS reduced MPW and TGW, ultimately abridged the grain yield. A decrease in GY of barley has been observed because of reduced fertile tillers and number of grains under drought conditions^69^. Likewise, Nawaz et al.^67^ and Fatemi et al.^70^ also reported a reduction in grain yield and associated parameters in wheat and barley, respectively, under short supply of water. A substantial enhancement in yield and contributing attributes was recorded by priming quinoa seeds with Se both in control and DS plants. The Se seed priming exhibits a beneficial impact on the growth and productivity of quinoa crop. Hajiboland et al.^68^ also reported similar finding that Se priming improved whole shoot and straw weight, spike and seed yield of wheat both under normal and DS conditions. Similarly, Sadak and Bakhoum^71^ also described an increase in chlorophyll contents, PCs, P, K, grain weight, BY and GY by Se application in quinoa. Se supplementation increases respiratory potential, metabolites accumulation, delays senescence^72,73^ and protect cells from stress induced apoptosis^74^ ultimately enhance crop yield. The biplot of PCA identified many significant factors, including stomatal conductance, osmotic potential, transpiration rate, leaf chlorophyll content and water use efficiency, as potent explanatory variables that were responsible for quinoa’s ability to withstand drought (Fig. 4) as also reported by Iqbal et al.^34^. Concludingly, the seed filling stage of quinoa was found most sensitive to drought stress due to limited water availability, reduced pollen viability and fertilization and compromised nutrient transport to developing seeds which reduced seed size, MPW, TGW, and ultimately diminished overall yield of quinoa. However, only the appropriate amount of Se shows positive response while higher levels of Se leads to toxicity by disrupting plant metabolic processes, leading to oxidative stress and cellular damage, inhibiting photosynthesis, impairing nutrient uptake and transport, ultimately impeding growth and reducing yield of quinoa plants^75^.

## Conclusion

Drought episodes during critical growth stages (flowering and seed filling) reduce overall plant performance, quality attributes and yield of quinoa. However, Se seed priming at varying concentrations demonstrated a remarkable potential to enhance drought tolerance and overall yield of quinoa. Notably, Se priming at 6.0 mg L^−1^ improved the growth, yield (MPL, MPW, TGW), physiological (LCC, PR, TR, SC) attributes, optimized water relations (LWP, LTP, LOP, LRWC, WUE) and grain quality of quinoa by reducing the deleterious effects of drought. Furthermore, seed filling stage of quinoa was identified as highly sensitive stage to DS and the seed priming with 6.0 mg L^−1^ Se could be used as an effective tool for sustainable quinoa production even under drought stress conditions.

## Data Availability

The datasets analysed during this study are included in this manuscript.
